# Validation of the Chinese Maudsley three-item visual analogue scale to measure depressive symptoms in a youth population

**DOI:** 10.1192/bjo.2024.778

**Published:** 2024-11-05

**Authors:** Yi Ding, Rebecca Strawbridge, Allan H. Young, Lingfeng Xue, Fangfang Zhao

**Affiliations:** School of Nursing and Rehabilitaiton, Nantong University, Nantong, China; Nantong Central Blood Station, Jiangsu Province, Nantong, China; Department of Psychological Medicine, Institute of Psychiatry, Psychology & Neuroscience, King's College London, London, UK; Department of Old Age Psychiatry, Institute of Psychiatry, Psychology & Neuroscience, King's College London, London, UK

**Keywords:** Depression, youth, visual analogue scale, reliability, validity

## Abstract

**Background:**

Existing self-rated depression measurement tools possess a range of psychometric drawbacks, spanning a range of validity and reliability constructs. The gold standard self-rated depression scales contain several variable items that are often non-specific, require respondents to have a certain level of language understanding and limited scoring options resulting in low sensitivity. The Maudsley three-item visual analogue scale (M3VAS) was developed to address these challenges.

**Aims:**

This study aimed to translate the M3VAS into Chinese and test its reliability and validity.

**Method:**

First, both M3VAS scales (assessing current severity and change in severity) were translated according to a standardised protocol to finalise the Chinese version. Reliability and validity were then examined among 550 young people with moderate to severe depression (patient health questionnaire-9 (PHQ-9) score ≥15) in a cross-sectional opportunistic questionnaire survey.

**Results:**

The content validity of each item (six items, across both scales) ranged from 0.83 to 1.00. Exploratory factor analysis denoted a total of two common factors, with a variance contribution rate of 64.34%. The total score correlated positively with the PHQ-9 total score (*r* = 0.241, *P* < 0.01). The Chinese version of the M3VAS had good reliability and validity values, and the confirmatory factor model fit well.

**Conclusions:**

The psychometric properties of the Chinese version of the M3VAS suggest that this scale can feasibly evaluate depression among young people in China.

Major depression is characterised by low mood and anhedonia, which can lead to functionally impairing cognitive and behavioural changes in patients. According to data from the World Health Organization (WHO), over 300 million people worldwide suffer from depression, with the illness considered the largest single cause of disability worldwide.^1^

## Youth is a high-risk period for depression

According to the ‘Medium and Long Term Youth Development Plan’ from China (2016–2025), the youth population is aged between 14 and 35 years old. Youth is a critical developmental period, and thus the promotion of physical and mental health is of great significance. Youth is also a period during which psychosocial stress can increase vulnerability to depression. In recent years, the prevalence of depression in the youth population has been increasing, which has wider consequences for the direct and indirect burden of this illness. Youth depression is, as such, a research priority.

## Common Depression Questionnaire

Commonly used depression severity measurement tools currently include the depression experience questionnaire (DEQ), children's self-rating depression scale (DSRSC), adolescent depression scale (RADS), hospital anxiety and depression scale (HADS) and children's depression scale (CDI), and those not specific to young people include the Hamilton depression scale (HRSD), self-rating depression scale (SDS), Beck depression inventory (BDI), depression state inventory (DSI) and Center for Epidemiologic Studies depression scale (CES-D). These tools possess a range of drawbacks: they contain several items, a high proportion of which are not common across scales;^2^ patient-rated tools are important for obtaining subjective experience but these are often not suitable for individuals with low-age reading abilities;^3^ each item also contains few scoring possibilities, which results in low variability and sensitivity.^4^

## The Maudsley three-item visual analogue scale (M3VAS)^4^

Compared to these tools, visual analogue scales have several advantages, including simpler and more intuitive completion; high resolution; lack of response restriction to predefined categories; and greater potential sensitivity to changes over time.^5^ However, previous visual analogue scales in the area of affect/emotion, such as the visual analogue mood scale,^6^ have only evaluated low mood as a single item, without measuring other core symptoms of depression (anhedonia), or including a specific duration of assessment (e.g. 2 weeks, which is necessary for conceptualising a depressive episode). The Maudsley three-item visual analogue scale (M3VAS)^4^ was developed in light of the aforementioned evidence, with items solely focused on the core symptoms of depression (low mood and anhedonia, plus suicidality as a core safety and severity construct) and using the visual analogue scale with its advantages of simplicity, intuition and high sensitivity. Further detail as to the original M3VAS scale have been outlined previously.^4^ At present, neither the M3VAS-current (assessment of symptom severity over the past 2 weeks) nor the M3VAS-change (assessment of change since last assessment/intervention) have been validated in languages other than English. Mandarin Chinese (including standard Chinese) has more native speakers than any other language globally and China is now a global leader in scientific research outputs.

This study therefore sought to first undertake a translation of the scale into standard Chinese and second report on its reliability and validity in a youth sample in China.

## Method

### Study design and participants

This study was a cross-sectional survey, employing convenience sampling methods to collect data among Chinese youth groups. The survey report was distributed with participants recruited through online advertising. Participants were provided monetary compensation for filling out questionnaires. The inclusion criteria were (a) young people aged between 14 and 35 years old and (b) willingness to participate in the study. These analyses were conducted for participants with a patient health questionnaire-9 (PHQ-9) score equal to or greater than 15. This study complied with the STROBE (strengthening the reporting of observational studies in epidemiology) statement.^7^ The STROBE statement refers to a list of items that should be included in observational study reports using three main designs in epidemiology: cohort design, case-control design and cross-sectional design. The research ethics committee of Nantong University approved this study (approval number 20220929-01). All participants in this study voluntarily participated and were required to sign an informed consent form before completing the survey. For participants under the age of 18, written informed consent was obtained from participants’ guardians.

### Cultural debugging

The Chinese version of the M3VAS was debugged according to the regulations on cultural debugging in the guidelines for cross-cultural debugging of the self-assessment scale.^8^ In this study, a total of 15 experts conducted two rounds of cultural adaptability research on the scale, adjusting for content that did not conform to Chinese language habits in the scale. Without violating the original scale, this included evaluation and revision of the meaning and expression of scale items to make the language conform to Chinese expression habits, resulting in a provisional version of the M3VAS in Chinese.

Expert inclusion criteria were as follows: (a) engaged in nursing, psychology and other related research for more than 10 years; (b) familiar with the use of measurement tools related to nursing, psychology and health; (c) having a senior professional title; (d) understand the research content and voluntarily participate. Among the 15 experts, five had a bachelor's degree, seven had a master's degree and three had a doctoral degree.

The scale was back translated (i.e. translated from Chinese to English, blind to the original English scale) by bilingual doctoral researchers (native Chinese academics working in England). The versions were then compared by the full team before being finalised.

### Measures

#### Social demographics

In the cross-sectional survey questionnaire, participant demographic data, including gender, age, alcohol use and smoking, were collected.

#### Maudsley three-item visual analogue scale

The M3VAS^4^ aims to operationally evaluate the current severity and change in severity of three core depressive symptoms (depression, loss of pleasure and suicide), rated by participants. Each item is scored using a 100 mm horizontal line used to indicate the range of possible responses, with descriptions at each end denoting the extreme values of the anchoring scale. Patients are asked to mark the most suitable location for their current state (M3VAS-current) or relevant changes they have experienced (M3VAS-change) in this case, both over the prior 2 weeks. Each item therefore scores on a 0–100 points range. In the M3VAS-current, 0 indicates no symptoms and 100 indicates extremely severe or frequent symptoms. In the M3VAS-change scale, the score range is from −50 to +50, where the negative end represents a negative change experienced by the patient since the start of the study or treatment (worsening), and the positive end represents a positive change (improvement). The total score of the scale is the value obtained by adding the scores of three items; the M3VAS-current therefore has a total score range of 0–300, and the M3VAS-change has a total score range between −150 and +150.^4^

#### Patient health questionnaire-9

The PHQ-9^9^ is an internationally recognised, widely used depression self-assessment scale. The scale comprises one item for each of the nine depressive symptoms from the DSM from 0 (completely absent) to 3 (almost every day). DSM is a classification and diagnostic standard for mental disorders developed by the American Psychiatric Association. The total score ranges from 0 to 27. Scores of 5, 10 and 15 are considered to represent mild, moderate and severe depression, respectively.^9,10^

### Statistics

IBM SPSS statistics was used for statistical analysis of the data. This study conducted statistical analysis of data using SPSSAU on the Wenjuanxing website. Descriptive statistics were used to present the demographic characteristics of participants. An item analysis of the scale was conducted using critical ratio and total item correlation methods. Content validity was analysed using the content validity index.^11^ Structural validity was analysed using exploratory factor analysis. Pearson's correlations compared total scores from the Chinese version of the M3VAS with the PHQ-9 to evaluate criterion validity. Confirmatory factor analysis (CFA) calculated the heterotrait-monotrait ratio of correlations (HTMT) value for discriminant validity, and the standard load coefficients for each item were calculated for aggregated validity. The average variances extracted (AVE) and composite reliability values for each item were calculated for aggregate validity analysis. Finally, the reliability of the scale was calculated by calculating Cronbach's α coefficient, the McDonald's ω coefficients and theta coefficients.

## Results

### Participant characteristics

A total of 550 people participated in this study. Among them, 367 women accounted for 67%, and 183 men accounted for 33%. Some 77% of participants were between the ages of 18 and 24, 89% of participants were unmarried, 64% of participants had siblings, 75% of participants did not drink alcohol and 86% of participants did not smoke. Please refer to Table 1 for details.

**Table 1:** Sociodemographic data (*N* = 550)

Variable	*N*	Percentage (%)
Gender		
Male	183	33.27
Female	367	66.73
Age		
14–18	22	4.00
18–24	423	76.91
25–29	80	14.55
30–35	25	4.55
Alcohol consumption		
Yes	139	25.27
No	411	74.73
Marital status		
Unmarried	489	88.91
Married	61	11.09
Smoke		
Yes	72	14.09
No	478	86.91

### Reliability and validity results

#### Critical ratio method

According to the definition and principles of the critical ratio method, the total score is arranged in order of high and low, with scores of 25−33% before and after being taken. The average value of the two groups is calculated, and the difference between the two averages is analysed, to provide the ‘critical ratio’.^12^ As such, the Chinese version of the M3VAS was sorted by total score and the top 27% (153 cases) of the scores were assigned to the high-severity group, while the bottom 27% (149 cases) were assigned to the low-severity group. Two independent-sample *t*-tests were then performed to compare the two sets of data (‘extreme group comparison’), as shown in Table 2.

**Table 2:** Project analysis results of the Chinese version the Maudsley three-item visual analogue scale

Entry	Extreme group comparison		Items related to total score	
	Decision value	*P*	*R*	*P*
1	12.150	<0.001	0.488	<0.001
2	12.770	<0.001	0.520	<0.001
3	16.603	<0.001	0.629	<0.001
4	12.323	<0.001	0.530	<0.001
5	10.939	<0.001	0.494	<0.001
6	10.607	<0.001	0.456	<0.001

#### Correlation analysis method

The correlation coefficient between the scores of each item in the Chinese version of the M3VAS and the total score of the scale (‘items related to total score’) ranged from 0.456 to 0.629 (*P* < 0.01), as shown in Table 2.

### Scale validity

#### Content validity

The content validity index was used to evaluate the M3VAS scales,^11^ and each expert evaluated the items in the scale separately. The content validity was evaluated using a four-point method, where ‘irrelevant’ scores 1 point, ‘somewhat relevant’ scores 2 points, ‘quite relevant’ scores 3 points and ‘highly relevant’ scores 4 points. The Content Validity Index (CVI) is divided into two categories: Item level Content Validity Index (I-CVI) and Scale level Content Validity Index (S-CVI). The results showed that the first round I-CVI values of the Chinese version of the M3VAS were 0.867–1.000, the scale content validity index/average (S-CVI/Ave) value was 0.933 and the scale content validity index/universal agreement (S-CVI/UA) value was 0.33. In the second round, the I-CVI values, S-CVI/Ave values and S-CVI/UA values were all 1.000. Full details of the two rounds of inquiries are specified in the supplementary appendix.

#### Construct validity

Exploratory factor analysis was conducted on the M3VAS scale.^14^ The Bartlett spherical test value of the M3VAS was 741.488 (*P* < 0.01), indicating that the scale could be used for factor analysis. The Kaiser–Meyer–Olkin value for M3VAS sampling was 0.650, indicating weak partial correlation between variables and good factor analysis suitability. Using the orthogonal rotation principal component analysis method, the commonality values corresponding to all study items were greater than 0.4, indicating a strong correlation between the study items and factors. Therefore, all six items across the two scale versions were retained. Two common factors (eigenvalues >1) were extracted in principal component analysis, with contribution rates of 32.47% and 31.87%, respectively. The load of each entry on the corresponding common factor was greater than 0.4, with the maximum value being 0.859 and the minimum value being 0.664. The factor composition matrix and factor naming are shown in Table 3.

**Table 3:** Exploratory factor analysis results of the Maudsley three-item visual analogue scale

Dimension	Entry	Change	Current
Current	1	−0.216	0.817
	2	−0.035	0.750
	3	0.041	0.815
Change	4	0.849	0.026
	5	0.859	−0.046
	6	0.664	−0.126
Eigenvalue		1.948	1.912
Variance contribution rate (%)		32.47	31.87
Cumulative variance contribution rate (%)		32.47	64.34

**Fig. 1:**
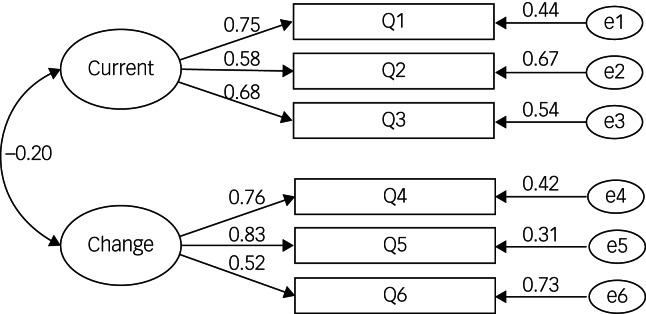
Confirmatory factor analysis model of the Chinese version the Maudsley three-item visual analogue scale.

The CFA model of the M3VAS is shown in Fig. 1. The values of each fitting indicator were displayed as follows: chi square value (χ^2^/d.f.) = 6.275, standardized root mean square residual (RMSEA) = 0.098, normalized fitting index (NFI)  = 0.933, non standardized fitting indicators (TLI) = 0.892, goodness of fit index (GFI) = 0.973, incremental fit index (IFI) = 0.943, comparative fit index (CFI) = 0.942.

#### Discriminant validity

This study used CFA to calculate the HTMT value for discriminant validity analysis. In the M3VAS, the HTMT value between the two common factors ‘state’ and ‘change’ was 0.219. We used exploratory factor analysis to calculate the AVE and composite reliability values of each dimension in the M3VAS for aggregate validity analysis, as shown in Table 4.

**Table 4:** Aggregation validity analysis of the Chinese version of the Maudsley three-item visual analogue scale

Variable	AVE	Critical ratio
Current	0.453	0.711
Change	0.513	0.753

AVE, average variances extracted.

#### Criterion-related validity

There was a significant positive correlation between the total score of the Chinese version of the M3VAS and the PHQ-9 score, with a correlation coefficient of 0.241 (*P* < 0.01).

### Scale reliability

- The reliability values (Cronbach's alpha and *ω* and theta coefficients) of the Chinese version of the M3VAS are shown in Table 5.
- One week after the assessment, 30 participants were randomly selected from the sample and the measure was repeated for the Cronbach's α retest reliability. The Chinese version of the M3VAS-current scale Cronbach's alpha coefficient was 0.756 and for the change scale it was 0.634.

**Table 5:** Reliability values of the Maudsley three-item visual analogue scale

Scale	Cronbach's *α* coefficient	*ω* coefficient	Theta coefficient
Current	0.709	0.841	0.716
Change	0.687	0.839	0.716

## Discussion

This study indicated promising reliability and validity of the Chinese versions of the M3VAS scale. In terms of validity, our findings suggested good discrimination between scale items (composite reliability value >3), correlations between item and total scores (Pearson's *r* > 3),^13^ content validity (I-CVI ≥ 0.78, S-CVI/UA ≥ 0.8 and S-CVI/Ave ≥ 0.9 in the second round),^11^ structural and construct validity (single factor per scale, contributing 63.34%), discriminant validity (HTMT < 0.9) and aggregate validity (AVE > 0.5 and composite reliability > 0.7). Reliability, particularly of the ‘current’ scale, appeared good (Cronbach's alpha >0.7).^15^

Content validity refers to the appropriate level of sampling of the content or behavioural range to be tested by the project. The values of I-CVI, S-CVI/UA and S-CVI/Ave are calculated based on the scores given by experts in the relevant field. The general standard is of good content validity when I-CVI ≥ 0.78, S-CVI/UA ≥ 0.8 and S-CVI/Ave ≥ 0.9.^11^ The results of this study showed that in the first round of the Chinese version of the M3VAS, except for the S-CVI/UA value, all other indicators met the requirements, with a S-CVI/UA value of 0.33. This finding may have been influenced by the consultation of several experts. Through expert inquiry and pre-experiment cultural debugging, the composition of the expert group was scientifically reasonable, and the positive coefficients of both rounds of expert inquiry achieved 100%. In the second round, the content validity indicators met the standards, indicating that the final Chinese version of the M3VAS had good content validity.

For construct validity, a total of two common factors were extracted, with each factor loading ranging from 0.664 to 0.859, and a cumulative contribution rate of 63.34%. The CFA fitting index values of the M3VAS were as follows: χ^2^/d.f. = 6.275, RMSEA = 0.098, NFI = 0.933, TLI = 0.892, GFI = 0.973, IFI = 0.943, CFI = 0.942. These values suggest overall that the model fits well, indicating good structural validity of the Chinese version of the M3VAS scale.

A reliability coefficient greater than 0.8 indicates very good reliability of the scale; greater than 0.7 reflects good reliability; greater than 0.6 can be acceptable; if the reliability coefficient is less than 0.6, a new scale needs to be developed.^14^ In this study, the Cronbach's α coefficient of the Chinese version of the M3VAS-current scale was 0.709, the ω coefficient was 0.841 and the theta coefficient was 0.716. For the M3VAS-change scale, the Cronbach's α coefficient was 0.687, the McDonald's ω coefficient was 0.839 and the theta coefficient was 0.716. All coefficient values were greater than 0.6, but results suggest that the current scale is more reliable than the change scale and may be a preferred outcome measure.

Some 30 cases were randomly selected from the sample to repeat the measure 1 week later, and the Cronbach's α coefficient was calculated on these. The results showed the Chinese version of the M3VAS-current scale to have a Cronbach's α coefficient of 0.756, and for the change scale the Cronbach's α coefficient was 0.634. These test–retest reliability parameters appear similar to above; the higher values for the current scale may indicate that to assess change over time, a repetition of the ‘current’ scale might hold more value than use of the change scale. However, a validation of the scale in an interventional longitudinal study is needed to establish reliability where changes are expected over time. Here, a detailed impression of severity at two timepoints would be obtained, timepoint benchmarks used in the change scale would be clear and standardised intervention would be delivered between these two timepoints.

We note the relatively low correlations between the M3VAS and the PHQ-9. While both are patient-rated scales, there may be several reasons for this, which could be established through further validation across different representative populations and particularly in specific interventional controlled trials. At this initial stage, we highlight two stark differences between these scales, in addition to the clear difference in the range of symptoms examined: first, that they use distinctly different scoring systems (with few categories per item versus wide-ranging continuous scores per item); and, second, that the PHQ-9 rates severity based on the *frequency* of symptoms rather than *acuteness*, while the M3VAS rates severity based on *acuteness* rather than *frequency* (or rather, does not specifically direct a rating based on frequency but leaves the participant themselves to account – or not account – for this aspect of severity). The PHQ-9 does also not have a specific ‘change’ scale.

Indeed, these preliminary findings suggest that the ‘change’ scale may be inferior to utilising the ‘current’ scale in repeated measures. The ‘change’ scale had been developed with a view to identifying whether patient-rated improvement, when explicitly requested, would differentiate from the common definition of improvement through using a single scale at multiple timepoints. A discrepancy between these measures could indicate a difference between how effective a participant believes an intervention has been; alternatively, it could illustrate a difference in recall bias or other phenomenological factor related to an individual's experience of a depressive episode. We believe that this could be established through further examination in interventional studies and/or through specific participant (qualitative) consultation, which this study was not able to do.

### Limitations

Because of the specific age group of the participants, our results may not generalise to all adult depression. In future research, the reliability and validity of the Chinese version of the M3VAS in other age groups could be further verified by expanding the sample age range, promoting the widespread application of the scale. This also applies to other aspects of generalisability, for example, this study did not ascertain a detailed classification of sample geographical sources. Participants were not independently evaluated by clinicians or researchers, and complex phenomena including psychiatric and non-psychiatric comorbidities were not measured. In the test–retest reliability examination, few participants were retested over a short interval of time. There were certain limitations with evaluating the ‘change’ scale, considering this was measured only at one point in time, and it is possible that participants rated this with variable ‘change’ timeframes in mind. In addition, it was impossible to know what changes had occurred in terms of intervention and psychosocial stress of the respondents during this period.

Although we adapted the scale for use in a different language, this does not consider cultural differences. Clearly, the PHQ-9 examines somatic in addition to ‘core’ symptoms of depression, and it is possible that people in China are more likely to rate more highly on somatic than other symptoms of depression.^16^ As above, further in-depth research can elucidate the applicability of this scale and depression severity across cultures.

The current research results indicated that the M3VAS had good validity and reliability in the Chinese youth population, and because of its relatively small number of items/written description, participants can easily complete it. We call for studies to validate this scale also in older adult, clinical and non-clinical, populations. As it stands, the M3VAS appears suitable for depression evaluation in the Chinese youth population. Its examination in interventional longitudinal studies will provide further information as to whether it is a sensitive measure of clinical outcome.

## Supporting information

Ding et al. supplementary material Ding et al. supplementary material

## Data Availability

Not all the data used in this study are publicly available due to containing sensitive information. However, data can be shared upon reasonable request to the corresponding author, F.Z.
