# Supplementary material for: Validation of the Chinese Maudsley three-item visual analogue scale to measure depressive symptoms in a youth population
Source: BJPsych Open. 2024 Nov 5;10(6):e194. doi: 10.1192/bjo.2024.778 (PMC11698190; doi:10.1192/bjo.2024.778)
Supplement: Ding et al. supplementary material [file S2056472424007786sup001.docx]

**Appendix**

**M3VAS 1st round expert rating and item content validity results**

| Entry | expert rating | | | | | | | | | | | | | | | Number of experts rated 3 or 4 | I-CVI |
| --- | --- | --- | --- | --- | --- | --- | --- | --- | --- | --- | --- | --- | --- | --- | --- | --- | --- |
|  | 1 | 2 | 3 | 4 | 5 | 6 | 7 | 8 | 9 | 10 | 11 | 12 | 13 | 14 | 15 |  |  |
| 1 | 2 | 3 | 3 | 3 | 3 | 4 | 3 | 3 | 4 | 3 | 4 | 3 | 4 | 3 | 2 | 13 | 0.867 |
| 2 | 3 | 3 | 4 | 2 | 3 | 4 | 2 | 3 | 4 | 3 | 4 | 3 | 4 | 3 | 4 | 13 | 0.867 |
| 3 | 3 | 3 | 4 | 3 | 4 | 4 | 3 | 4 | 4 | 3 | 4 | 3 | 4 | 3 | 4 | 15 | 1.000 |
| 4 | 3 | 3 | 4 | 3 | 4 | 3 | 3 | 4 | 4 | 3 | 4 | 3 | 4 | 4 | 4 | 15 | 1.000 |
| 5 | 4 | 3 | 4 | 3 | 4 | 4 | 2 | 4 | 4 | 3 | 4 | 3 | 4 | 3 | 4 | 14 | 0.933 |
| 6 | 4 | 3 | 3 | 4 | 2 | 4 | 3 | 4 | 4 | 3 | 4 | 3 | 3 | 3 | 4 | 14 | 0.933 |

| entry | expert rating | | | | | | | | | | | | | | | Number of experts rated 3 or 4 | I-CVI |
| --- | --- | --- | --- | --- | --- | --- | --- | --- | --- | --- | --- | --- | --- | --- | --- | --- | --- |
|  | 1 | 2 | 3 | 4 | 5 | 6 | 7 | 8 | 9 | 10 | 11 | 12 | 13 | 14 | 15 |  |  |
| 1 | 4 | 3 | 3 | 3 | 3 | 4 | 3 | 3 | 4 | 4 | 4 | 3 | 3 | 4 | 3 | 15 | 1.000 |
| 2 | 4 | 3 | 3 | 3 | 3 | 4 | 3 | 3 | 4 | 4 | 4 | 3 | 3 | 4 | 3 | 15 | 1.000 |
| 3 | 4 | 3 | 4 | 3 | 3 | 4 | 4 | 3 | 4 | 3 | 4 | 4 | 4 | 4 | 3 | 15 | 1.000 |
| 4 | 4 | 3 | 4 | 3 | 3 | 4 | 4 | 4 | 4 | 3 | 4 | 3 | 4 | 4 | 3 | 15 | 1.000 |
| 5 | 4 | 4 | 4 | 3 | 3 | 3 | 4 | 4 | 4 | 3 | 4 | 3 | 4 | 4 | 3 | 15 | 1.000 |
| 6 | 3 | 4 | 4 | 3 | 4 | 3 | 4 | 4 | 4 | 3 | 4 | 3 | 4 | 4 | 4 | 15 | 1.000 |

**M3VAS 2nd round expert rating and item content validity results**
